# In response to “Randomized controlled trial of traxi panniculus retractor use during non‐emergent cesarean delivery”

**DOI:** 10.1002/pmf2.70469

**Published:** 2026-10-31

**Authors:** Jacqueline C. Hairston

**Affiliations:** ^1^ Department of Obstetrics and Gynecology Northwestern University Feinberg School of Medicine Chicago Illinois USA

The authors of “Randomized controlled trial of traxi panniculus retractor use during non‐emergent cesarean delivery” [1] aim to address the value of retractors in the surgical care of patients with obesity and yet what becomes abundantly clear is the need to divorce the body mass index (BMI) from body composition.

For far too long, obesity has been simplified to one number, BMI. The recent Lancet Diabetes & Endocrinology Commission defined clinical obesity and detailed the way in which this condition may manifest in a patient's life, including body composition and distribution of adiposity [2]. Accordingly, a study evaluating the impact of a panniculus retractor on surgical duration should look beyond BMI and include the panniculus grade.

In the plastic surgery literature, Iglesias et al. detail a classification system that grades the redundant pannus, from 1 to 5, based on its location relative to the inguinal ligament, thigh, and knee while a patient is standing [3]. This system has been used in the obstetrics literature. Wise et al. modified the grading system by assessing the panniculus while study participants were supine, and a grade 0 was added to indicate the absence of a panniculus. In their study, pregnant patients with a BMI of 40 or higher were randomized to have their anatomy scan conducted with or without a panniculus retractor. Of the 171 patients who consented to participate, 20 (11.7%) were found to be Grade 0 (no panniculus) and were thus excluded from the study. Furthermore, while they found no difference in the average BMI for their study groups (control: 46.2 kg/m^2^ | retractor: 46.7 kg/m^2^), the sole statistically significant imbalance detected in the baseline characteristics for their randomized controlled study was the panniculus grade. A total of 65.8% of the participants in the control arm had a grade 1 panniculus compared to 45.9% of the participants in the retractor arm. No participants had a Grade 4 or 5 panniculus [4]. These two findings highlight how BMI is underpowered to characterize habitus.

In figure 1, Anwer et al. acknowledge that one patient in the TRAXI group was found not to have a retractable panniculus [1]. The authors explain the potential mechanisms by which mobilization of the panniculus could reduce the forced vital capacity; however, we have no information on whether a participant's panniculus was just below their inguinal ligament or below their knees. This is critical when assessing surgical duration as well as patient and provider sentiments surrounding the C‐section and retraction. Other limitations in the interpretation of this study are the lack of data on the type of skin incision performed and the high rate of medical taping (64.7%) in the no‐traxi arm.

Ongoing efforts to improve the care of patients living with obesity should be applauded; however, we must continue to think critically about the role of BMI in research.

## CONFLICT OF INTEREST STATEMENT

J.C.H. owns 0.33 shares in GE Healthcare.
